# Carbapenemase Producing Bacteria in the Food Supply Escaping Detection

**DOI:** 10.1371/journal.pone.0126717

**Published:** 2015-05-12

**Authors:** Beverly J. Morrison, Joseph E. Rubin

**Affiliations:** Department of Veterinary Microbiology, University of Saskatchewan, Saskatoon, Saskatchewan Canada; University of Calgary, CANADA

## Abstract

Carbapenem antimicrobials are critically important to human health and they are often the only remaining effective antibiotics for treating serious infections. Resistance to these drugs mediated by acquired carbapenemase enzymes is increasingly encountered in gram-negative bacteria and is considered a public health emergency. Animal origin food products are recognized as a potential source of resistant organisms, although carbapenem resistance has only recently been reported. In western countries there are active resistance surveillance programs targeting food animals and retail meat products. These programs primarily target beef, pork and poultry and focus exclusively on *E*. *coli*, *Salmonella*, *Campylobacter* spp. and *Enterococcus* spp. This global surveillance strategy does not capture the diversity of foods available nor does it address the presence of resistance gene-bearing mobile genetic elements in non-pathogenic bacterial taxa. To address this gap, a total of 121 seafood products originating in Asia purchased from retail groceries in Canada were tested. Samples were processed using a taxa-independent method for the selective isolation of carbapenem resistant organisms. Isolates were characterized by phenotypic antimicrobial susceptibility testing, PCR and DNA sequencing. Carbapenemase producing bacteria, all *bla*_OXA-48_, were isolated from 4 (3.3%) of the samples tested. Positive samples originated from China (n=2) and Korea (n=2) and included squid, sea squirt, clams and seafood medley. Carbapenemase producing organisms found include *Pseudomonas*, *Stenotrophomonas* and *Myroides* species. These findings suggest that non-pathogenic bacteria, excluded from resistance surveillance programs, in niche market meats may serve as a reservoir of carbapenemase genes in the food supply.

## Introduction

Antimicrobial resistance is a serious threat to the future of antimicrobial chemotherapy, and negatively impacts the health of humans and animals. Food is recognized as a potential source of resistant organisms, although the magnitude of the negative impact of these bacteria on human health is controversial and often politicized. Consequently, governments have implemented resistance surveillance programs targeting food and designed to detect the emergence of resistance. These programs provide the requisite empiric data for human health risk assessment, to inform policy and aid in the prudent use of antimicrobials. These programs are largely Anglo/Euro centric, targeting meats such as pork, poultry and beef while ignoring the diversity of other terrestrial or aquatic species consumed globally (Table 1). There is also notable pathogen centricity, with an exclusive focus on zoonotic or potentially pathogenic indicator organisms such as *Salmonella*, *Campylobacter E*. *coli* and *Enterococcus* spp. (Table 1) [1–4]. The use of indicator organisms like *E*. *coli* may be an efficient strategy for identifying resistance among bacteria in cattle, pigs and poultry where *E*. *coli* recovery rates are nearly 100%, but not as effective when evaluating other meats (seafood for example) with different and ill-defined microbial communities [2,5]. When testing seafood, the World Organization for Animal Health (OIE) recommends including *Vibrio* spp. or *Aeromonas* isolates, however these foodstuffs are not routinely captured by most surveillance programs (Table 1) [1,5]. Although a pathogen specific approach provides rational, discrete targets for resistance surveillance, this narrow highly specific focus may fail to identify resistance in the broader microbial community. In a landmark 2014 report entitled "Antimicrobial Resistance: Global Report on Surveillance", the World Health Organization suggested the need for an integrated One-Health oriented approach for antimicrobial resistance surveillance [6]. Key gaps, including a lack of understanding of the movement of antimicrobial resistant organisms or resistance genes into people from the food chain, and the role of the global food systems in the dissemination of resistance were identified [6].

**Table 1:** Summary of global resistance surveillance programs targeting food animal and retail meat bacterial isolates.

Country	Year-Program	Animal / Products	Bacterial Species	Website
**North America**				
**Canada**	2011-CIPARS	cattle, pig, chicken	*Salmonella*, *Campylobacter*, *E*. *coli*	http://www.phac-aspc.gc.ca/cipars-picra/index-eng.php
**USA**	2011-NARMS	cattle, pig, chicken, turkey	*Salmonella*, *Campylobacter*, *E*. *coli*, *Enterococcus*	http://www.fda.gov/animalveterinary/safetyhealth/antimicrobialresistance/nationalantimicrobialresistancemonitoringsystem/default.htm
**Europe**				
**Denmark**	2012-DANMAP	cattle, pig, chicken, milk,	*Salmonella*, *Campylobacter*, *E*. *coli*, *Enterococcus*, *C*. *difficile*, MRSA (*S*. *aureus*)	http://www.danmap.org/
**Finland**	2007–09-FINRES-VET	cattle, pig, chicken, turkey, sheep, eggs	*Salmonella*, *Campylobacter*, *E*. *coli*, *Enterococcus*, MRSA (*S*. *aureus*)	http://www.evira.fi/portal/en/about+evira/publications/?a=category&cid=28
**Italy**	2003-ITAVARM	cattle, pig, chicken, turkey, milk, molluscs,	*Salmonella*, *E*. *coli*, *Enterococcus*	http://195.45.99.82:800/pdf/itavarm.pdf
**Netherlands**	2013-MARAN	cattle, pig, chicken, turkey, dairy cattle, veal calves, lamb	*Salmonella*, *Campylobacter*, *E*. *coli*, *Enterococcus*	http://www.wageningenur.nl/en/Research-Results/Projects-and-programmes/MARAN-Antibiotic-usage.htm
**Norway**	2012-NORM-VET	cattle, pigs, chicken, wild reindeer	*Salmonella*, *E*. *coli*, *Enterococcus*, MRSA (*S*. *aureus*)	http://www.vetinst.no/eng/Publications/NORM-NORM-VET-Report
**Sweden**	2013-SVARM	cattle, pig, chicken, turkey, lamb	*Salmonella*, *Campylobacter*, *E*. *coli*, *Enterococcus*	*www*.*sva*.*se/en/Antibiotika/SVARM-reports/*
**Asia/Pacific**				
**Australia**	2007-Pilot Surveillance Program for Antimicrobial Resistance in Bacteria of Animal Origin	cattle, pig, chicken	*Campylobacter*, *E*. *coli*, *Enterococcus*	http://www.daff.gov.au/agriculture-food/food/regulation-safety/antimicrobial-resistance
**Japan**	2007-JVARM	cattle, pig, chicken	*Salmonella*, *Campylobacter*, *E*. *coli*, *Enterococcus*, *S*. *aureus*	http://www.maff.go.jp/nval/tyosa_kenkyu/taiseiki/monitor/e_index.html

The animal and bacterial species targeted by each surveillance program were extracted from the most recently available report. The website from which the report was downloaded is listed in the right hand column. CIPARS—Canadian Integrated Program for Antimicrobial Resistance Surveillance, NARMS—National Antimicrobial Resistance Monitoring System, DANMAP—Danish Integrated Antimicrobial Resistance Monitoring and Research Programme, FINRES-VET The Finnish Veterinary Antimicrobial Resistance Monitoring and Consumption of Antimicrobial Agents report, ITAVARM—Italian Veterinary Antimicrobial Resistance Monitoring, MARAN—Monitoring of Antimicrobial Resistance and Antibiotic Usage in Animals in the Netherlands, NORM-VET Norwegian Surveillance System for Antimicrobial Drug Resistance, SVARM—Swedish Veterinary Antimicrobial Resistance Monitoring, JVARM—The Japanese Veterinary Antimicrobial Resistance Monitoring System.

Intercontinental travel has globalized the problem of antimicrobial resistance [7,8]. Resistant organisms and resistance-conferring genes are not regionally confined; this mobility is exemplified by the global dissemination of the NDM and KPC carbapenemases from initial hotspots in the Indian sub-continent and northeastern United States respectively [7]. International travel is now recognized as an important risk factor for infection with these organisms [8,9]. Unlike human travel, the role of the global food trade in the dissemination of carbapenemase genes has not been investigated. The dissemination of carbapenem resistance occurs with the spread of resistant organisms, but is also facilitated by mobile genetic elements such as plasmids or integrons [10–12]. It is therefore crucial to focus on the presence of resistance genes in microbial communities rather than simply the presence of resistance in particular taxa.

The scale of the global seafood trade is enormous. In 2008 it comprised ~39% of world seafood production (live weight) and was valued at over $107 billion US [13]. Despite the importance of aquatic and marine species to food security and the economy, seafood products are conspicuously absent from the majority of resistance surveillance programs (Table 1). Asia is the world's leading aquaculture region, including the top 6 producing countries and 3 of the top 10 exporters [13]. Trade of farmed species which may be exposed to antibiotics during production for disease control, or resistance genes or organisms bearing them in agricultural or sewage effluents may unintentionally result in the export of resistant organisms or genes.

Importation of seafood products from endemic regions of Asia into countries with a low prevalence of carbapenemase producers, led us to hypothesize that these products may be an unrecognized source of carbapenemase genes [7]. Carbapenem resistance is truly a public health emergency, in 2013 the U.S. Centers for Disease Control and Prevention classified carbapenem resistance (specifically among the Enterobacteriaceae) as an "urgent threat" requiring immediate action [14]. Previous studies have documented the presence of carbapenemase producing organisms in rivers, sewage effluents and agricultural animals including pigs, poultry and dairy cows [15]. In a small pilot study including only six food samples, we identified a VIM-2 carbapenemase producing *Pseudomonas fluorescens*-like organism isolated from a squid imported to Canada from Korea [16]. The objective of this larger follow-up study was to determine if this isolate was anomalous or indicative of a broader phenomenon.

## Materials and Methods

### Samples collection and processing

A convenience sample of 121 frozen meat and seafood items imported from China (n = 58), Vietnam (n = 22), Korea (n = 13), Thailand (n = 8), Taiwan (n = 8), India (n = 6), Philippines (n = 4) and Japan (n = 2) were purchased from 17 groceries in Vancouver (n = 51), 3 in Saskatoon (n = 20) and 10 in the Toronto (n = 50) census metropolitan areas in Canada. Products from cephalopod (n = 26), tunicate (n = 2), bivalve (n = 21) and gastropod mollusk (n = 5), crustacean (n = 18), cnidarian (n = 1), anuran (n = 10), chelonian (n = 5), a variety of finned fish species (n = 31) and seafood medley products (n = 3) were included.

A taxa-independent phenotypic screening strategy for carbapenemase-producing organisms, derived from methods used by North American surveillance programs was used [2]. Frozen samples were transported to Saskatoon in coolers by overnight courier. A 25g portion of the sample was dissected and washed with vigorous agitation in 250ml of buffered peptone water in a sterile, single-use sample bag. The rinsate was then incubated overnight at 35°C. Following incubation, a 25μL aliquot was plated onto Mueller-Hinton agar containing 2μg/ml meropenem for the selective isolation of carbapenem-resistant organisms as described by the CLSI [17]. Any organisms growing were then gram stained, and gram-negatives were identified by PCR and sequencing of the *cpn*60 or 16S genes [18–20].

### Antimicrobial susceptibility testing and carbapenemase gene detection and identification

Antimicrobial minimum inhibitory concentrations were determined by the broth micro-dilution and gradient strip methods using the NARMS Sensititre plates and E-tests. Testing was done in accordance with the Clinical and Laboratory Standards Institute (CLSI) guidelines and manufacturer's instructions [21]. Categorical interpretive criteria (susceptible, intermediate and resistant) were not applied as CLSI approved breakpoints are not published for all organisms [17,22]. Raw MIC data is therefore presented.

Isolates were screened for the most clinically concerning acquired carbapenemase genes (VIM, IMP, NDM, KPC and OXA type) by single-plex PCR using previously published primers [18]. DNA sequences were deposited in the NCBI GenBank under the accession numbers (KM670111-4; KM670318 and KM677187-9). For carbapenemase producing isolates, the location of carbapenemase genes on plasmids was assessed using a broth conjugation model as previously described with the sodium azide resistant *E*. *coli* strain J53 as a plasmid recipient[8,23].

## Results and Discussion

Of the 121 samples tested, no bacteria producing VIM, IMP, NDM or KPC type carbapenemases were found in the present study. OXA-48 carbapenemase-producing organisms including *Stenotrophomonas*, *Myroides* and *Pseudomonas* spp. were isolated from four samples (Table 2). Meropenem and ertapenem MICs for these isolates ranged from 1.5 - > 32μg/ml and 0.75 - >32μg/ml respectively and all isolates were uninhibited by the majority of β-lactams tested (Table 2). The seafood medley and clam isolates were notable for their elevated colistin, kanamycin and tetracycline MICs, while the squid isolate was uninhibited by trimethoprim + sulfamethoxazole and chloramphenicol (Table 2). Interestingly, the ciprofloxacin MICs of all isolates were low (≤2) falling below the CLSI resistance breakpoint for "other non-Enterobacteriaceae"[17].

**Table 2:** Source, identification and antimicrobial susceptibility of organisms producing carbapenemases.

Seafood Product	Seafood Medley*	Clams	Sea Squirt	Squid
**Market Location**	Saskatoon	Saskatoon	Vancouver	Toronto
**Country of Origin**	China	China	Korea	Korea
**Bacterial Species**	*Stenotrophomonas maltophila*	*Myroides odoratimimus*	*Stenotrophomonas* spp.	*Pseudomonas putida*
***bla* Gene**	OXA-48	OXA-48	OXA-48	OXA-48
**E-test MIC (μg/ml)**				
**Ertapenem**	>32	0.75	>32	3
**Meropenem**	>32	3	>32	1.5
**Tigecycline**	1	1.5	0.38	3
**Colistin**	>256	16	0.5	1
**Sensititre MIC (μg/ml)**				
**Ampicillin**	>32	16	>32	>32
**Amox + Clav** §	32	8	>32	>32
**Cefoxitin**	>32	>32	>32	>32
**Ceftriaxone**	>64	>64	64	32
**Ceftiofur**	>8	>8	>8	>8
**Naladixic Acid**	>16	>16	2	0.5
**Ciprofloxacin**	2	2	2	0.25
**Kanamycin**	>64	>64	≤8	≤8
**Streptomycin**	≤32	>64	≤32	≤32
**Gentamicin**	8	16	4	32
**Azithromycin**	>16	1	8	>16
**Chloramphenicol**	8	16	8	>32
**Tetracycline**	32	>32	8	≤4
**SXT** ‡	0.25	2	0.5	>4

^§^ amoxicillin + clavulanic acid.

^‡^trimethoprim + sulfamethoxazole.

*Seafood medley contains squid, octopus, mussels and shrimp.

The carbapenemase-producing organisms identified are neither common food-borne nor animal pathogens and are not used as indicators of resistance and therefore would not have been identified by current resistance surveillance programs. Although transfer of the phenotype was not observed following attempts at broth conjugation, suggesting a chromosomal location of the OXA-48 gene, additional studies are required to determine whether or not this gene is located in a mobile genetic element. The presence of carbapenemase genes in bacteria not considered clinically relevant, and living in meats not sampled in resistance surveillance programs, has the potential to allow these genes to enter the food supply undetected.

While cooking to an adequate internal temperature (70°C for fin fish and 74°C for other seafoods) would almost certainly kill carbapenemase producing organisms, cultural preferences for eating raw seafood (Japanese sashimi or Korean Hoe for example) may provide a unique opportunity for the transmission of antimicrobial resistant organisms or their genes [24]. The carbapenems are critically important antimicrobials for treating infections with multi-drug resistant gram-negative bacteria. The emergence and dissemination of carbapenemase producing organisms is therefore a serious threat to public health and one of the gravest dangers to the future of antimicrobial chemotherapy [15,25].

Including the previous report, carbapenemase producing organisms were isolated from 5 of 127 (4%) products tested, indicating that these organisms may not be anomalous in seafood [16]. Although our investigation relied on a convenience sampling and was not designed to identify high risk products or countries of origin, only foods from Korea and China tested positive for these organisms and products containing squid were over represented. The epidemiology of carbapenemase producing organisms in foods clearly requires definition and additional study. Furthermore, to define the significance of these of these bacteria to human health, the location of these genes (chromosomally or on mobile genetic elements) should be determined.
